# Dengue Virus Serotype 3 Origins and Genetic Dynamics, Jamaica

**DOI:** 10.3201/eid3010.240170

**Published:** 2024-10

**Authors:** Shanice A. Redman, Lester J. Perez, Kenn Forberg, Keisha Francis, Jerome P. Walker, Tamara K. Thompson, Heather Phillips, Gavin A. Cloherty, Michael G. Berg, Joshua J. Anzinger

**Affiliations:** Abbott Pandemic Defense Coalition, Kingston, Jamaica (S.A. Redman, K. Francis, J.P. Walker, T.K. Thompson, H. Phillips, J.J. Anzinger);; The University of the West Indies, Kingston (S.A. Redman, K. Francis, J.P. Walker, T.K. Thompson, H. Phillips, J.J. Anzinger);; Abbott Laboratories, Abbott Park, Illinois, USA (L.J. Perez, K. Forberg, G.A. Cloherty, M.G. Berg);; Abbott Pandemic Defense Coalition, Abbott Park (L.J. Perez, K. Forberg, G.A. Cloherty, M.G. Berg);; Global Virus Network, Baltimore, Maryland, USA (J.J. Anzinger)

**Keywords:** dengue, dengue virus, dengue virus serotype 3, sequence, serotype, genotype, vector-borne infections, viruses, zoonoses, Caribbean, Jamaica

## Abstract

We identified 3 clades of dengue virus serotype 3 belonging to genotype III isolated during 2019–2020 in Jamaica by using whole-genome sequencing and phylogenomic and phylogeographic analyses. The viruses likely originated from Asia in 2014. Newly expanded molecular surveillance efforts in Jamaica will guide appropriate public health responses.

An estimated 390 million dengue virus (DENV) infections occur each year worldwide (*1*), and cases are expected to increase because of vector expansion (*2*). In the Americas, dengue incidence has increased dramatically since the 1990s (*3*), yet molecular epidemiologic investigations of DENV have remained uncommon for many countries in the region.

Molecular surveillance of DENV in Jamaica has been limited, and serotype testing has been typically performed only for select samples during epidemics. DENV genomic sequencing has rarely been performed in the Caribbean and almost never in Jamaica; only 1 published study from Jamaica analyzed sequences of patient samples collected in 2007 (*4*). Jamaica was established as an Abbott Pandemic Defense Coalition site in 2022 (*5*) and next-generation sequencing (NGS) was introduced in response to the COVID-19 pandemic, so NGS virus surveillance is becoming routine.

In 2019, after a brief lull in dengue cases in the Americas after the introduction of Zika virus (ZIKV) in 2015–2016, a massive surge in dengue cases occurred throughout the region (*3*,*6*), at which time Jamaica recorded its greatest number of dengue cases (6). We describe DENV whole-genome NGS results for patients who sought clinical care in Jamaica for dengue during the 2018–2020 epidemic and examine DENV transmission dynamics by using phylogenetics and phylogeography.

## The Study

We obtained residual diagnostic serum samples positive for DENV nonstructural (NS) protein 1 that were collected from patients at the University Hospital of the West Indies in Kingston, Jamaica, during December 2019–September 2020. Fourteen of 15 total samples were collected during December 2019–January 2020, when cases exceeded the Jamaica Ministry and Health and Wellness’s epidemic threshold; the additional serum sample was collected at the end of the epidemic during September 2020 (*7*). We extracted total nucleic acids from samples by using the Abbott Diagnostics *m*2000sp instrument (Abbott, https:/www.abbott.com) and performed virus RNA enrichment by using the Comprehensive Viral Research Panel probe set (Twist Bioscience, https://www.twistbioscience.com). 

**Figure 1:**
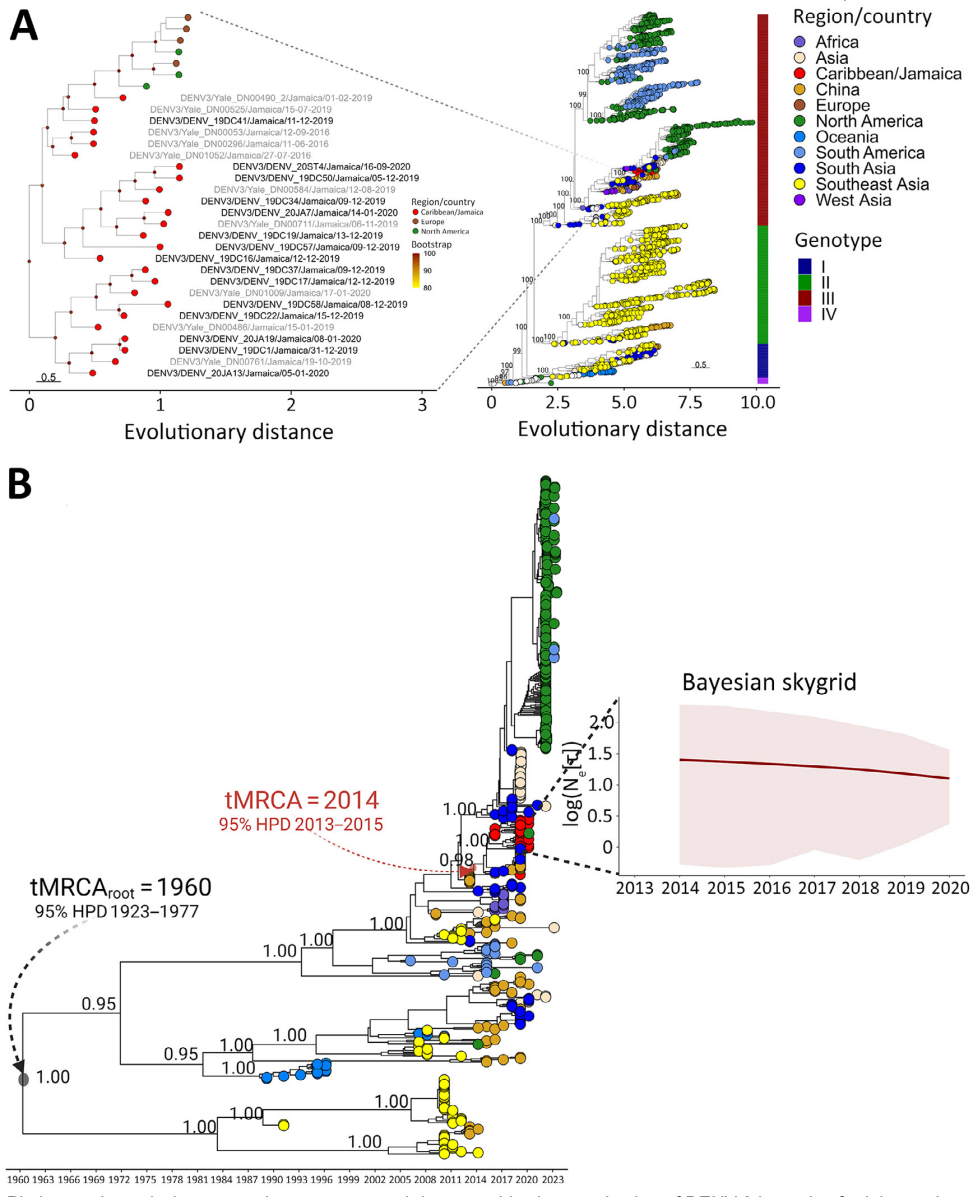
Phylogenetic analysis, temporal emergence, and demographic characterization of DENV-3 in study of origins and genetic dynamics, Jamaica. A) Maximum-likelihood phylogenetic trees of global DENV-3 sequences indicate the strains circulating in Jamaica belong to genotype III and are organized into 3 independent clades (left tree). The sequences were mapped according to the country of sampling and their genotypes (right tree) by using the *ggtreeExtra* R package (The R Project for Statistical Computing, https://www.r-project.org). Bootstrap values >80% are displayed for all nodes in the left tree (colored circles at nodes) and only for external nodes and main clade of interest in the main tree (right side). All bootstrap values are shown in Appendix Figure 1. Scale bars indicate nucleotide substitutions per site. B) Time-scaled maximum clade credibility tree of DENV-3 sequences indicates the temporal emergence of DENV-3 strains in Jamaica starting in 2014. tMRCA and 95% HPD intervals for the tree root and the clade containing the sequences from Jamaica are indicated. Confidence values, determined by posterior probabilities, are indicated for external nodes and the node of interest. Full node support for the tree is indicated in Appendix Figure 2, panel A. Bayesian skygrid plot of the effective population size (Ne[τ]) over time indicates median values and 95% HPD intervals. DENV-3, dengue virus serotype 3; HPD, highest posterior density; tMRCA, time to most recent common ancestor.

We obtained 5 whole (100% coverage), 7 near-whole (91%–99% coverage), and 3 partial (28%–65% coverage) genome sequences (Appendix Table 1); all were DENV serotype 3 (DENV-3). We retrieved all DENV-3 sequences available in Nextstrain (https://nextstrain.org) and aligned them with sequences and metadata from this study (https://github.com/LesterJP/Dengue_Jamaica_Study) by using MAFFT (*8*). We inferred maximum-likelihood phylogenetic reconstructions by using IQ-TREE2 (Appendix). Sequences from Jamaica formed a monophyletic clade within DENV-3 genotype III (GIII), which was further divided into 3 monophyletic subclades: 2 exclusively containing sequences from Jamaica and 1 containing sequences from Jamaica, North America, and Europe (Figure 1, panel A). Those results suggested DENV-3 circulation in Jamaica might have been either from multiple introductions or from endemic evolution that later mirrored the genetics of other DENVs in global circulation. A temporal analysis traced the emergence of DENV-3 to 1960, which had an evolutionary rate of 3.96 × 10^−3^ substitutions/site/year; DENV-3 GIII was most likely introduced into Jamaica during 2014 (Figure 1, panel B). A Bayesian skygrid reconstruction of the DENV-3 monophyletic clade from Jamaica (Appendix) revealed that genetic diversity remained stable, and the evolutionary rate was 1.78 × 10^−3^ substitutions/site/year.

**Figure 2:**
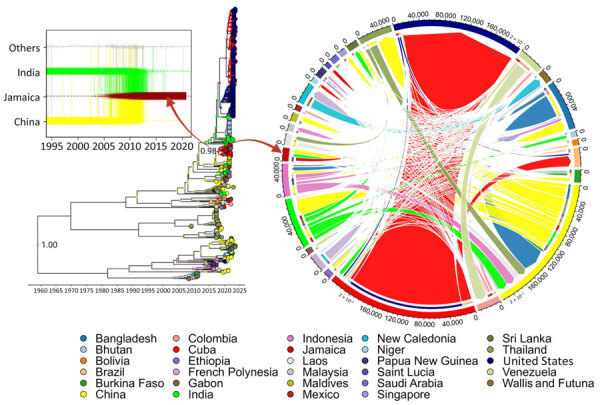
Time-scaled explicit discrete phylogeographic analysis of dengue virus serotype 3 (DENV-3) spread in Jamaica. Relationship between the dispersal trajectory of DENV-3 in the maximum clade credibility tree and the specific DENV-3 migration patterns into Jamaica over time (box) is indicated. Nodes of the tree represent the inferred country of origin for sampled strains. Arrows indicate the nodes from which taxa were selected for analysis by using the TaxaMarkovJumpHistoryAnalyzer (https://github.com/beast-dev/beast-mcmc). Only values of discrete state probability for the root and the node of interest are shown in the tree; complete state probability values of the nodes are indicated in Appendix Figure 2, panel B. Dynamic pathways of DENV-3 geographic movement are indicated by Markov jump mappings (right circular map). Transmission network of DENV-3 is summarized by Markov jump events, analyzed using TreeMarkovJumpHistoryAnalyzer and visualized in a circular layout by using the circlize package in R (The R Project for Statistical Computing, https://www.r-project.org). The width of each link reflects the frequency of virus movement; quantitative estimates were provided by using the TreeMarkovJumpHistoryAnalyzer. Tick marks on the outside of the circle’s segments indicate virus movement frequencies.

We conducted discrete phylogeographic analyses to determine whether DENV-3 genetic diversification in Jamaica arose from external introductions or endemic circulation (Figure 2). Integrating country-specific data with virus dispersal trajectories identified Indonesia as the putative origin of DENV-3 (i.e., the tree root) and showed a clear pattern of intercountry virus spread. A Markov jump reward plot showed the intracountry and intercountry dynamics of DENV-3 globally, indicating China, India, Thailand, and Bangladesh were key virus exporters with high interconnectivity. In the Caribbean, Cuba was a primary source for regional DENV-3 spread and introduction into the United States. For Jamaica specifically, this analysis and the TaxaMarkovJump history reconstruction method (*9*) indicated 2 major concurrent importation events from Asia; strains from Jamaica were subsequently transmitted to Saint Lucia and other potentially unsampled Caribbean countries. Further intracountry diversification likely led to the 2018–2020 dengue epidemic.

**Figure 3:**
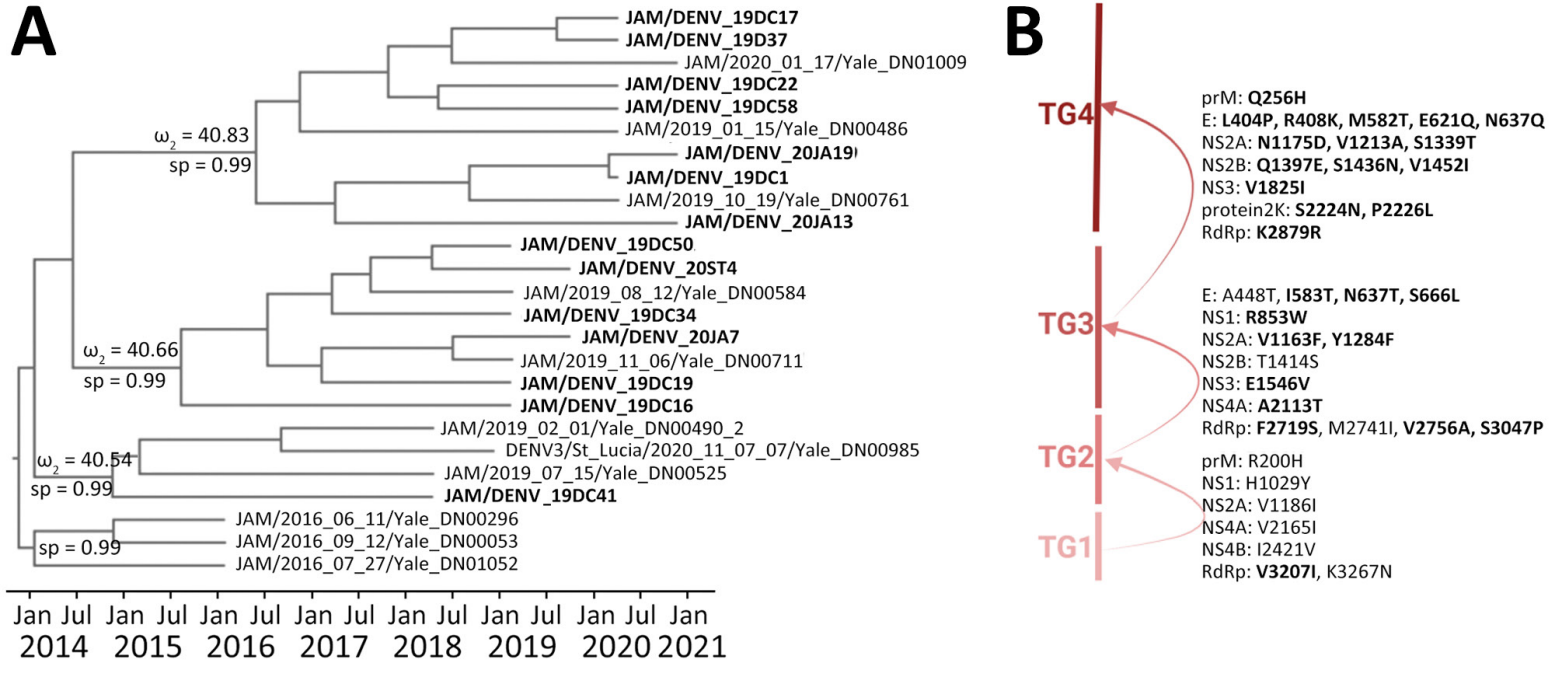
Time-scaled phylogenetic analysis, molecular characterization, dynamics, and natural selection of dengue virus serotype 3 in Jamaica. A) Phylogenetic tree indicates monophyletic clusters of strains from Jamaica (bold text) extracted from the discrete phylogeographic analysis (Figure 2). Discrete sp values (ω) for nodes evaluated for episodic selection are shown. Full sp values for nodes are shown in Appendix Figure 2, panel B. B) Strains were evaluated for amino acid replacements according to each TG. Arrows indicate episodic selection of each main TG clade. Bold text indicates positively selected mutations. sp, state probability; TG, temporal group.

**Figure 4:**
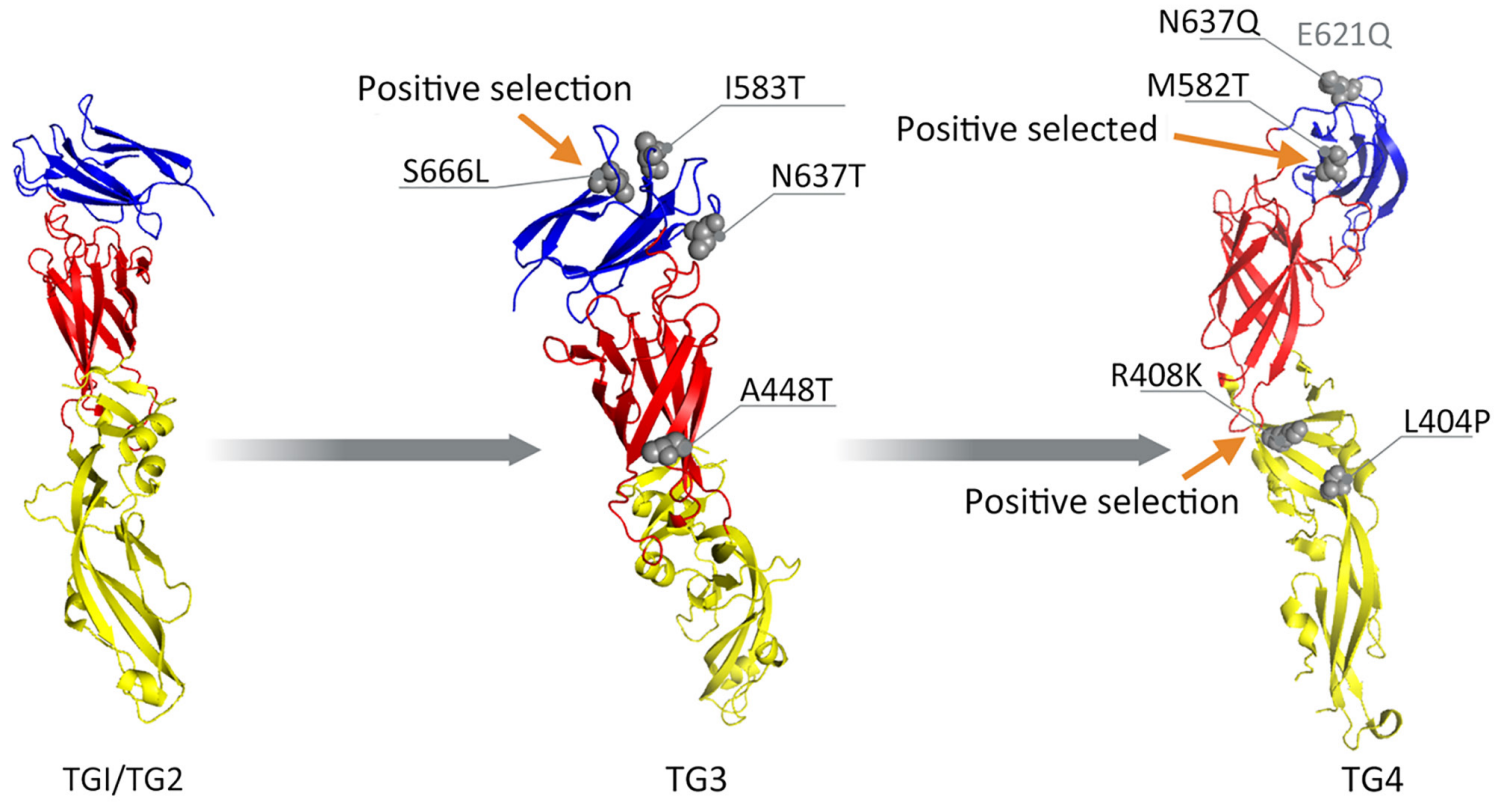
Envelope glycoprotein 3-dimensional structures (structure 7a3s; RCSB Protein Data Bank, https://www.rcsb.org) from dengue virus serotype 3 strains in Jamaica. Red indicates protein domain I, yellow indicates domain II, and blue indicates domain III. Gray spheres indicate mutations identified across various TGs. Arrows indicate mutations detected by site models. E621Q (faded text) is in the loop region not visible in the crystal structure. TG, temporal group.

To analyze mutation signatures associated with temporal clades, we extracted strains from Jamaica from the discrete phylogeographic tree and categorized them into temporal groups (TGs) (Figure 3, panel A). Initially (TG1→TG2), amino acid replacements were infrequent and dispersed throughout NS genes. Over time (TG2→TG3), an accumulation of mutations was concentrated in the RNA-dependent RNA polymerase and envelope (E) genes. This trend continued (TG3→TG4) with additional mutations in E and NS2 genes. Mutations in the E gene were located within the lateral ridge and hinge epitopes of strains in TG3 and in the lateral ridge epitope for TG4 (Figure 3, panel B; Figure 4). This pattern of mutations, particularly in the E gene that encodes the most critical protein target of neutralizing antibodies (*10*), suggests that immune evasion contributed to selection of those mutations. In contrast, we found no indication of immune evasion for E gene domain III (receptor-binding protein) and fusion peptide sequences; those regions were not identified under positive selection.

Episodic and pervasive selection analyses to assess sudden and consistent positive selection were performed to determine which DENV-3 strains and mutations were evolutionarily favored. Branch-site models applied to the complete DENV-3 coding sequence indicated the emergence of each successive time-epoch lineage was under positive, episodic selection (Appendix Table 2). This selection was the predominant force driving retention of numerous mutations within key functional domains for virus replication and structural proteins and determining the evolutionary trajectory of those strains (Figure 3, panel A). We applied site models for the E protein to ascertain which mutations were subject to positive, pervasive selection (Appendix Tables 3, 4) and were likely causes of immune escape. Domain III mutations S666L in TG3 and M582T in TG4 and domain I/II mutation R408K in TG4 strains located at the hinge region were positively selected residues (Figure 3, panel B; Figure 4).

Introductions from Asia most likely brought DENV-3 to Jamaica in 2014, and it was first detected in the country in 2016 (https://www.who.int/emergencies/disease-outbreak-news/item/4-february-2019-dengue-jamaica-en). A mixed outbreak of DENV-3 and DENV-4 infections occurred in 2016 when ZIKV was detected (*11*). Our findings show that DENV-3 GIII continued to circulate at low levels in Jamaica after 2016, which then led to an explosive DENV-3 GIII disease epidemic during November 2018–March 2020. It remains inconclusive why a period of low-level DENV circulation occurred in the Americas after the introduction of ZIKV in 2015–2016, although ZIKV cross-immunity with DENV and public health interventions and behavioral changes in response to ZIKV might have contributed (*3*,*12*).

The E glycoprotein is a critical target of neutralizing antibodies (*10*). Our findings indicated E glycoprotein mutations were positively selected, underscoring their relevance for adaptive evolutionary responses that might have influenced strain prevalence and virulence. Neutralizing antibodies against E protein domain III of DENV-3 GIII are typically weaker than those against other domains (*13*), which might explain why domain III mutations were observed over time in our study; subneutralizing antibodies would enable continued virus circulation. Those mutations are not expected to have a large effect on circulation because DENV immunity usually protects from homotypic reinfection; however, breakthrough infections have been described and could contribute to sustained DENV circulation (*14*).

## Conclusions

The circulation of DENV-3 during 2014–2020, a dengue outbreak in 2016, and a large dengue epidemic during 2018–2020 likely produced substantial population immunity to DENV-3 in Jamaica, which might have led to the introduction of a new DENV serotype(s), a well-described risk factor for severe dengue (*15*). Newly expanded molecular surveillance efforts in Jamaica will enable whole-genome NGS of DENV in clinical samples collected after 2020 to determine ongoing circulation patterns and guide appropriate public health responses.

Appendix Additional information for dengue virus serotype 3 origins and genetic dynamics, Jamaica.

## References

[1] Guzman MG, Gubler DJ, Izquierdo A, Martinez E, Halstead SB. Dengue infection. Nat Rev Dis Primers. 2016;2:16055. 10.1038/nrdp.2016.55 27534439

[2] Kraemer MUG, Reiner RC Jr, Brady OJ, Messina JP, Gilbert M, Pigott DM, et al. Publisher Correction: Past and future spread of the arbovirus vectors *Aedes aegypti* and *Aedes albopictus.* Nat Microbiol. 2019;4:901. 10.1038/s41564-019-0440-7 30962571 PMC7609323

[3] Perez F, Llau A, Gutierrez G, Bezerra H, Coelho G, Ault S, et al. The decline of dengue in the Americas in 2017: discussion of multiple hypotheses. Trop Med Int Health. 2019;24:442–53. 10.1111/tmi.13200 30624838 PMC6850595

[4] Brown MG, Salas RA, Vickers IE, Heslop OD, Smikle MF. Molecular epidemiology of dengue in Jamaica dengue virus genotypes in Jamaica, 2007. West Indian Med J. 2011;60:120–5. 21942113

[5] Averhoff F, Berg M, Rodgers M, Osmanov S, Luo X, Anderson M, et al. The Abbott Pandemic Defense Coalition: a unique multisector approach adds to global pandemic preparedness efforts. Int J Infect Dis. 2022;117:356–60. 10.1016/j.ijid.2022.02.001 35134559 PMC8817457

[6] Lue AM, Richards-Dawson MEH, Gordon-Strachan GM, Kodilinye SM, Dunkley-Thompson JAT, James-Powell TD, et al. Severity and outcomes of dengue in hospitalized Jamaican children in 2018–2019 during an epidemic surge in the Americas. Front Med (Lausanne). 2022;9:889998. 10.3389/fmed.2022.889998 35801209 PMC9254731

[7] Ministry of Health and Wellness, National Epidemiology Unit, Jamaica. Weekly epidemiology bulletin. December 12, 2020 [cited 2024 Jan 19]. https://www.moh.gov.jm/wp-content/uploads/2020/12/Weekly-Bulletin-EW-50_2020-.pdf

[8] Rozewicki J, Li S, Amada KM, Standley DM, Katoh K. MAFFT-DASH: integrated protein sequence and structural alignment. Nucleic Acids Res. 2019;47(W1):W5–10. 10.1093/nar/gkz342 31062021 PMC6602451

[9] Hong SL, Lemey P, Suchard MA, Baele G. Bayesian phylogeographic analysis incorporating predictors and individual travel histories in BEAST. Curr Protoc. 2021;1:e98. 10.1002/cpz1.98 33836121 PMC8672455

[10] Gallichotte EN, Baric RS, de Silva AM. The molecular specificity of the human antibody response to dengue virus infections. Adv Exp Med Biol. 2018;1062:63–76. 10.1007/978-981-10-8727-1_5 29845525

[11] Anzinger JJ, Mears CD, Ades AE, Francis K, Phillips Y, Leys YE, et al.; ZIKAction Consortium1,2. ZIKAction Consortium1,2. Antenatal seroprevalence of Zika and chikungunya viruses, Kingston Metropolitan Area, Jamaica, 2017–2019. Emerg Infect Dis. 2022;28:473–5. 10.3201/eid2802.211849 35076369 PMC8798668

[12] Pan American Health Organization. Response to the epidemic of Zika virus in the Americas [cited 2024 Jan 19]. https://www.paho.org/sites/default/files/2019-04/Zika-Annual-Report-Dec-2015-2016.pdf

[13] Young E, Carnahan RH, Andrade DV, Kose N, Nargi RS, Fritch EJ, et al. Identification of dengue virus serotype 3 specific antigenic sites targeted by neutralizing human antibodies. Cell Host Microbe. 2020;27:710–724.e7. 10.1016/j.chom.2020.04.007 32407709 PMC7309352

[14] Waggoner JJ, Balmaseda A, Gresh L, Sahoo MK, Montoya M, Wang C, et al. Homotypic dengue virus reinfections in Nicaraguan children. J Infect Dis. 2016;214:986–93. 10.1093/infdis/jiw099 26984144 PMC5021223

[15] Guzman MG, Alvarez M, Halstead SB. Secondary infection as a risk factor for dengue hemorrhagic fever/dengue shock syndrome: an historical perspective and role of antibody-dependent enhancement of infection. Arch Virol. 2013;158:1445–59. 10.1007/s00705-013-1645-3 23471635

