# Supplementary material for: Dengue Virus Serotype 3 Origins and Genetic Dynamics, Jamaica
Source: Emerg Infect Dis. 2024 Oct;30(10):2149–54. doi: 10.3201/eid3010.240170 (PMC11431914; doi:10.3201/eid3010.240170)
Supplement: Appendix — Additional information for dengue virus serotype 3 origins and genetic dynamics, Jamaica. [file 24-0170-Techapp-s1.pdf]

Article DOI: https://doi.org/10.3201/eid3010.240170

EID cannot ensure accessibility for supplementary materials supplied by authors. Readers who have difficulty accessing supplementary content should contact the authors for assistance.

Dengue Virus Serotype 3 Origins and Genetic Dynamics, Jamaica

Appendix

Additional Methods

Sample Collection

Serum samples from patients seeking clinical care at the UHWI and testing positive for dengue virus nonstructural protein 1 (NS1) antigen were examined in this study. The sample set was obtained from storage at −20°C and spanned from December 2019 to September 2020. Dengue NS1 antigen positivity was determined by using either a Standard E Dengue NS1 Ag ELISA (SD Biosensor, https://www.sdbiosensor.co.in) or Captia Dengue NS1 ELISA (Trinity Biotech, https://www.trinitybiotech.com) according to the manufacturers' instructions. Information related to patient age, sex, and date of sample collection were all obtained from the UHWI laboratory information system. All samples were assigned a study identifier to maintain patient confidentiality.

Total Nucleic Acid Extraction and cDNA Synthesis

Samples were pretreated with benzonase (>250 units/µL; Sigma-Aldrich, https://www.sigmaaldrich.com) for 3 hours at 37°C to decrease human background nucleic acids (718 µL serum + 80 µL of 10× buffer + 2 µL benzonase). Samples with less than the required volume were diluted with 1% phosphate buffered saline to obtain the required 718 µL sample volume. Extraction of total nucleic acids from 500 µL of treated sample was performed on the Abbott Diagnostics *m*2000sp system by using the Sample Preparation System DNA (both Abbott Diagnostics, https:/www.abbott.com). The extraction contained a positive control (5 virus stocks spiked into HIV-positive plasma at 3.0-log copies/mL) and 1 negative control (normal human plasma). The cDNA was generated from 10 µL of extracted total nucleic acids by using the SuperScript IV First-Strand Synthesis System (ThermoFisher Scientific,

https://www.thermofisher.com) and Sequenase Version 2.0 DNA Polymerase (ThermoFisher Scientific). The products were cleaned by using EMnetik PCR cleanup beads (Beckman Coulter, https://www.beckman.com).

Library Preparation, Target Enrichment, and DNA Sequencing

Next-generation sequencing libraries were prepared by using the Illumina Nextera XT Library Preparation Kit (Illumina, https://www.illumina.com) for 24 cycles with nonbiotinylated, unique dual index barcode adapters (Integrated DNA Technologies, https://www.idtdna.com). Libraries were purified by using EMnetik PCR cleanup beads. Libraries were assessed for size and quality by using a 4200 TapeStation (Agilent, https://www.agilent.com) and quantified by using a Qubit Flex Fluorometer (ThermoFisher Scientific). Next, an aliquot of each metagenomic library (maximum of 24 libraries and 3,000 ng total) was pooled together for target enrichment. Pools were dried by using a vacuum centrifuge, resuspended in a solution of human Cot-1 DNA and Universal Nextera Blockers (Integrated DNA Technologies), and hybridized to Comprehensive Viral Research Panel (Twist Biosciences, https://www.twistbioscience.com) probes for 16 hours according to the manufacturer's instructions. Hybridized reads were captured by affinity interaction on streptavidin beads (Twist Biosciences), amplified by using a KAPA library amplification kit (Roche, https://www.roche.com), and repurified by using magnetic beads (Twist Biosciences). Virus-enriched libraries were analyzed for size and concentration as previously described. Next-generation sequencing of Comprehensive Viral Research Panel–enriched libraires was performed on an Illumina MiSeq instrument by using a MiSeq v2 300 cycle flow cell (Illumina). The resulting raw data were imported into CLC Genomics Workbench version 22 (QIAGEN, https://www.qiagen.com), reads were mapped to reference sequences of all 4 dengue serotypes, and consensus sequences were generated. In addition, an in-house pipeline (reVamp) that uses the Bowtie 2 aligner (https://bowtie-bio.sourceforge.net) was used to ensure consensus sequences were similar to those generated by the CLC Genomics Workbench.

Genomic Data Collection and Phylogenetic Analysis

We retrieved all sequences of DENV-3 available in the Nextstrain repository (https://github.com/nextstrain/dengue) on September 1, 2023. All downloaded sequences were aligned with the sequences obtained in this study by using MAFFT v7.453 (*1*) and settings for localpair option alignment. The aligned sequences were then used for maximum-likelihood (ML) phylogenetic inference by using IQ-TREE2 (*2*) as previously described (*3*).

Temporal and Demographic Bayesian Inference

To determine the temporal emergence and evolutionary rate of DENV-3 globally and for the strains circulating in Jamaica, time scaled phylogenies were generated by using BEAST 1.10.5 (*4*), and the BEAGLE 3 library (*5*) was used to enhance computational efficiency. The molecular clock and parameters used in this study have been previously described (*6*). To estimate the cutoff for the grid number, we performed an evaluation by using the skyride prior model, which yielded an initial evolutionary rate of 1.3×10^{-3} substitutions/site/year. The number of points obtained were used to select a skygrid tree prior model (*7*) for analysis. For each genomic segment, 8 independent runs were executed, each sampling from the Markov chain Monte Carlo chains. The total number of generations (states/chain length) of chains per run was 9×10^8; each run was sampled every 9×10^5 generations (steps). Convergence was determined by an effective sample size parameter estimate >200, calculated by using Tracer 1.7 software (*8*).

Discrete Phylogeographic Analysis

To explore the geographic dynamics of DENV-3 and determine the sources of external introductions and local emergence of lineages in Jamaica, a discrete trait phylogeographic inference (*9*) analysis was conducted as previously described (*3*). In addition, post hoc analyses to determine the MarkovJump estimates for transition histories were generated by using the new BEAST tree sampling tools TaxaMarkovJumpHistoryAnalyzer and TreeMarkovJumpHistoryAnalyzer (https://github.com/beast-dev/beast-mcmc). The R packages (The R Project for Statistical Computing, https://www.r-project.org) MarkovJumpR and circlize were used for visualization purposes.

Positive Selection Analyses

To analyze the effects of both pervasive and episodic selection on the evolution of DENV-3 GIII strains in Jamaica, we used previously described methods (*10*). Our approach involved 2 separate codon-based alignments. The first encompassed the entire polyprotein coding sequence, and the second focused solely on the envelope protein in the strains from Jamaica. Those alignments along with the time-stamped maximum clade credibility tree from our temporal analysis were used as inputs for the CODEML program within PAML v.4.9 software (*11*). To estimate episodic selection, we applied branch-site models to detect the emergence of lineages under positive selection within the complete coding genome of the strains from Jamaica. Each branch-site test on prespecified branches (foreground branches) were

compared with the remaining branches (background branches) by using the alternative model A and the null model A1. For branch-site likelihood ratio tests (LRTs), the null model was chosen as a simplified version of the selection model with fewer parameters, which was expected to fit the data less accurately (lower ML). The significance of the LRTs was determined by assuming that twice the difference in log ML between the 2 models followed a χ^2 distribution with degrees of freedom equal to the difference in the number of parameters between the models (Appendix Table 2). For assessing pervasive selection of the envelope protein, we used site models M2 and M8. The Empirical Bayes method was used to calculate posterior probabilities for sites under positive selection. To reduce false positives, we contrasted models M2 and M8, which identify positive-selection pressure, with models M1 and M7, indicative of neutral selection (*12*). Only LRTs that had significant results ($p<0.01$) were considered (Appendix Table 3, 4).

References

1. Rozewicki J, Li S, Amada KM, Standley DM, Katoh K. MAFFT-DASH: integrated protein sequence and structural alignment. Nucleic Acids Res. 2019;47:W5–10. PubMed https://doi.org/10.1093/nar/gkz342

2. Nguyen LT, Schmidt HA, von Haeseler A, Minh BQ. IQ-TREE: a fast and effective stochastic algorithm for estimating maximum-likelihood phylogenies. Mol Biol Evol. 2015;32:268–74. PubMed https://doi.org/10.1093/molbev/msu300

3. Perez LJ, Orf GS, Berg MG, Rodgers MA, Meyer TV, Mohaimani A, et al. The early SARS-CoV-2 epidemic in Senegal was driven by the local emergence of B.1.416 and the introduction of B.1.1.420 from Europe. Virus Evol. 2022;8:veac025. PubMed https://doi.org/10.1093/ve/veac025

4. Suchard MA, Lemey P, Baele G, Ayres DL, Drummond AJ, Rambaut A. Bayesian phylogenetic and phylodynamic data integration using BEAST 1.10. Virus Evol. 2018;4:vey016. PubMed https://doi.org/10.1093/ve/vey016

5. Ayres DL, Cummings MP, Baele G, Darling AE, Lewis PO, Swofford DL, et al. BEAGLE 3: improved performance, scaling, and usability for a high-performance computing library for statistical phylogenetics. Syst Biol. 2019;68:1052–61. PubMed https://doi.org/10.1093/sysbio/syz020

6. Perez LJ, Forberg K, Cloherty GA, Berg MG. Temporal and coevolutionary analyses reveal the events driving the emergence and circulation of human mamastroviruses. Emerg Microbes Infect. 2023;12:2217942. PubMed https://doi.org/10.1080/22221751.2023.2217942

7. Hill V, Baele G. Bayesian estimation of past population dynamics in BEAST 1.10 using the skygrid coalescent model. Mol Biol Evol. 2019;36:2620–8. PubMed https://doi.org/10.1093/molbev/msz172

8. Rambaut A, Drummond AJ, Xie D, Baele G, Suchard MA. Posterior summarization in Bayesian phylogenetics using Tracer 1.7. Syst Biol. 2018;67:901–4. PubMed https://doi.org/10.1093/sysbio/syy032

9. Lemey P, Hong SL, Hill V, Baele G, Poletto C, Colizza V, et al. Accommodating individual travel history and unsampled diversity in Bayesian phylogeographic inference of SARS-CoV-2. Nat Commun. 2020;11:5110. PubMed https://doi.org/10.1038/s41467-020-18877-9

10. Orf GS, Perez LJ, Meyer TV, Luk K-C, Forberg K, Rodgers MA, et al. Purifying selection decreases the potential for Bangui orthobunyavirus outbreaks in humans. Virus Evol. 2023;9:vead018. PubMed https://doi.org/10.1093/ve/vead018

11. Yang Z. PAML 4: phylogenetic analysis by maximum likelihood. Mol Biol Evol. 2007;24:1586–91. PubMed https://doi.org/10.1093/molbev/msm088

12. Anisimova M, Yang Z. Multiple hypothesis testing to detect lineages under positive selection that affects only a few sites. Mol Biol Evol. 2007;24:1219–28. PubMed https://doi.org/10.1093/molbev/msm042

Appendix Table 1. Sample information and sequencing metrics for dengue virus samples sequenced in this study*

Sample ID	GenBank accession no.	Date collected	Age, y/sex	Case classification	Total reads	Mapped reads	Consensus length†	Genome coverage, %†	Genome coverage, %‡
DENV_19DC1	PP902575	2019 Dec 31	48/F	Dengue + warning signs	971,542	735,000	10,603	99	99
DENV_19DC16	NA	2019 Dec 12	17/M	Dengue + warning signs	132,764	36,000	6,914	65	68
DENV_19DC17	PP902573	2019 Dec 12	45/M	Dengue + warning signs	277,388	62,000	9,772	91	93
DENV_19DC19	PP902571	2019 Dec 13	6/F	Dengue + warning signs	234,582	152,000	9,998	93	94
DENV_19DC22	PP902576	2019 Dec 15	20/F	NA	629,734	314,000	10,551	99	99
DENV_19DC34	PP902577	2019 Dec 9	59/F	Dengue + warning signs	2,629,532	1.7×10^6	10,632	99	99
DENV_19DC37	PP902578	2019 Dec 9	22/F	Dengue + warning signs	1,139,812	790,000	10,708	100	100
DENV_19DC41	PP902574	2019 Dec 11	3/F	NA	334,930	175,000	10,661	100	100
DENV_19DC50	PP902579	2019 Dec 05	21/F	NA	562,628	266,000	10,445	98	98
DENV_19DC57	NA	2019 Dec 9	12/NA	Dengue + warning signs	128,138	733	2,988	28	13
DENV_19DC58	NA	2019 Dec 8	18/M	Dengue + warning signs	46,312	9,553	3,893	36	33
DENV_20JA7	PP902580	2020 Jan 14	28/M	Dengue + warning signs	6,402,074	4.7×10^6	10,657	100	100
DENV_20JA13	PP902581	2020 Jan 5	7/M	Dengue + warning signs	115,956	780,000	10,218	95	97
DENV_20JA19	PP902582	2020 Jan 8	32/F	Dengue + warning signs	2,044,864	1.6×10^6	10,708	100	99
DENV_20ST4	PP902572	2020 Sep 16	38/F	NA	2,127,068	187,000	10,708	100	100

*ID, identification; NA, not available; +, positive.
†Consensus sequences and genome coverage of reads were determined by using the CLC Genomics Workbench version 22 (QIAGEN, https://www.qiagen.com).
‡An in-house pipeline (reVamp) that uses the Bowtie 2 aligner (https://bowtie-bio.sourceforge.net) was used to ensure consensus sequences were similar to those generated by CLC Genomics Workbench.

Appendix Table 2. PAML branch-site model A analysis to identify branches under positive, episodic selection in dengue virus serotype 3 genotype III strains in Jamaica*

Foreground	Null model		Alternative model			Positively selected
branches	Parameters	–lnL	Parameters†	–lnL	–2ΔlnL†	sites‡
Clade TG2	P_0 = 0.92588<br>P_1 = 0.07410<br>P_{2a} = 0.00002<br>P_{2b} = 0. 00001<br>ω_o = 0. 0001<br>ω_1 = 1.0000<br>ω_2 = 1.0000	20,490.1693	P_0 = 0.92626<br>P_1 = 0.07210<br>P_{2a} = 0.00152<br>P_{2b} = 0.00012<br>ω_o = 0. 0001<br>ω_1 = 1.0000<br>ω_2 = **40.5415**	20,476.77008	**26.8**	**3207**
Clade TG3	P_0 = 0.77062<br>P_1 = 0.10121<br>P_{2a} = 0.11329<br>P_{2b} = 0.01488<br>ω_o = 0. 0001<br>ω_1 = 1.0000<br>ω_2 = 1.0000	23,195.15758	P_0 = 0.92487<br>P_1 = 0.06535<br>P_{2a} = 0.00914<br>P_{2b} = 0.00065<br>ω_o = 0. 0001<br>ω_1 = 1.0000<br>**ω_2 = 40.655294**	20,227.37752	**5935.5**	147, **583**, **637**, **666**, **853**, **1163**, 1185, **1284**, 1486, **1546**, 1786, 1795, 1797, 1799, **2113**, 2165, 2394, 2421, 2452, 2459, 2462, 2467, 2468, **2719**, **2756**, **3047**
Clade TG4	P_0 = 0.58485<br>P_1 = 0.22459<br>P_{2a} = 0.13769<br>P_{2b} = 0.05288<br>ω_o = 0. 0001<br>ω_1 = 1.0000<br>ω_2 = 1.0000	34,773.9393	P_0 = 0.69797<br>P_1 = 0.15572<br>P_{2a} = 0.11962<br>P_{2b} = 0.02669<br>ω_o = 0. 02134<br>ω_1 = 1.0000<br>ω_2 = **40.831445**	20,476.77008	**28594.34**	**256, 404, 408,** 448, 526, 530, 539, 582, 621, 1014, 1015, 1016, 1017, 1018, 1019, 1024, 1026, 1027, 1028, 1029, 1030, 1033, 1038, 1047, 1173, **1175**, 1177, 1178, 1180, 1182, 1183, 1184, 1203, **1213, 1339, 1397, 1436, 1452, 1825,** 1832, 1833, 1989, **2224, 2226,** 2430, 2431, 2439, 2441, 2445, 2446, 2447, 2454, 2455, 2456, 2457, 2461, 2463, 2464, 2466, 2470, 2509, 2535, 2536, 2562, 2588, 2741, **2879**

*PAML v.4.9 software (*11*) was used for phylogenetic analysis. –lnL, highest log-likelihood (probability) of the phylogeny; P, site class in distribution; TG, temporal group; ω, state probability.
†p<0.01, χ^2 = 5.99. Bolded text indicates statistical significance (ω_2>1 and –2ΔlnL>5.99).
‡Bold text indicates positively selected mutation sites in strains from Jamaica.

Appendix Table 3. Positively selected sites in the envelope gene of dengue virus serotype 3 and parameters estimated by the CODEML program in the PAML package*

Model	log-likelihood score	Parameters	Positive sites
M1	−2204.036947	P_0 = 0.55151, P_1 = 0.44849	NA
M2	−2191.113835	ω_2 = 21.322802, P_0 = 0.43491,P_1 = 0.54966, P_2 = 0.01543	408, 666
M7	−2204.112964	p = 0.00501, q = 0.00747	NA
M8	−2191.155276	ω_2 = 19.574365, P_0 = 0.98447, p = 0.00500, q = 0.00500, P_1 = 0.01553	408, 582, 666

*PAML v.4.9 software (*11*) was used for phylogenetic analysis. Codons were selected that had a posterior probability >0.99 belonging to the positively selected class (ω>1). NA, not applicable; P, site class in distribution; p and q, beta distribution parameters; ω, state probability.

Appendix Table 4. Statistical analysis to avoid false estimations of sites under positive selection pressure*

Gene	Models compared	−2Δl†	df	dN/dS‡
Envelope	M1 versus M2	**25.86**	2	21.32
	M7 versus M8	**25.9**	2	19.57

*Neutral models (M1 and M7) were compared with selection models (M2 and M8). Δl, likelihood-ratio statistic; df, degrees of freedom between nested models.
†p<0.01, χ^2 = 9.21. Bold text indicates statistical significance (–2ΔlnL>9.21).
‡Ratio of nonsynonymous to synonymous substitutions.

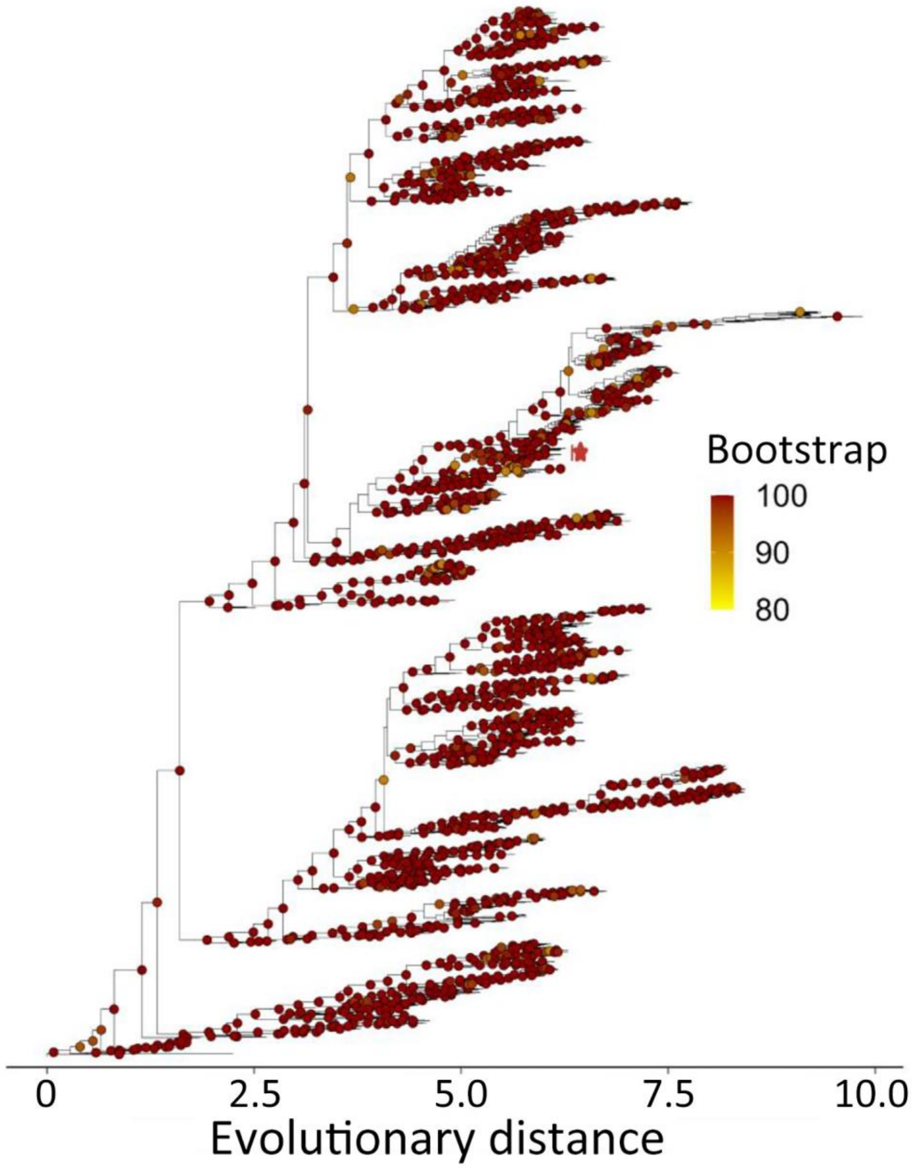

Appendix Figure 1. Maximum-likelihood phylogenetic analysis of dengue virus serotype 3 (DENV-3) isolates from Jamaica. All DENV-3 genomes available in Nextstrain (https://github.com/LesterJP/Dengue_Jamaica_Study) were used to reconstruct the genotypic demarcation topology and internal node support. Bootstrap values are scaled according to color from 80% (yellow) to 100% (red); values <80% are not displayed. Red star indicates the specific clade where strains from Jamaica are located in the tree.

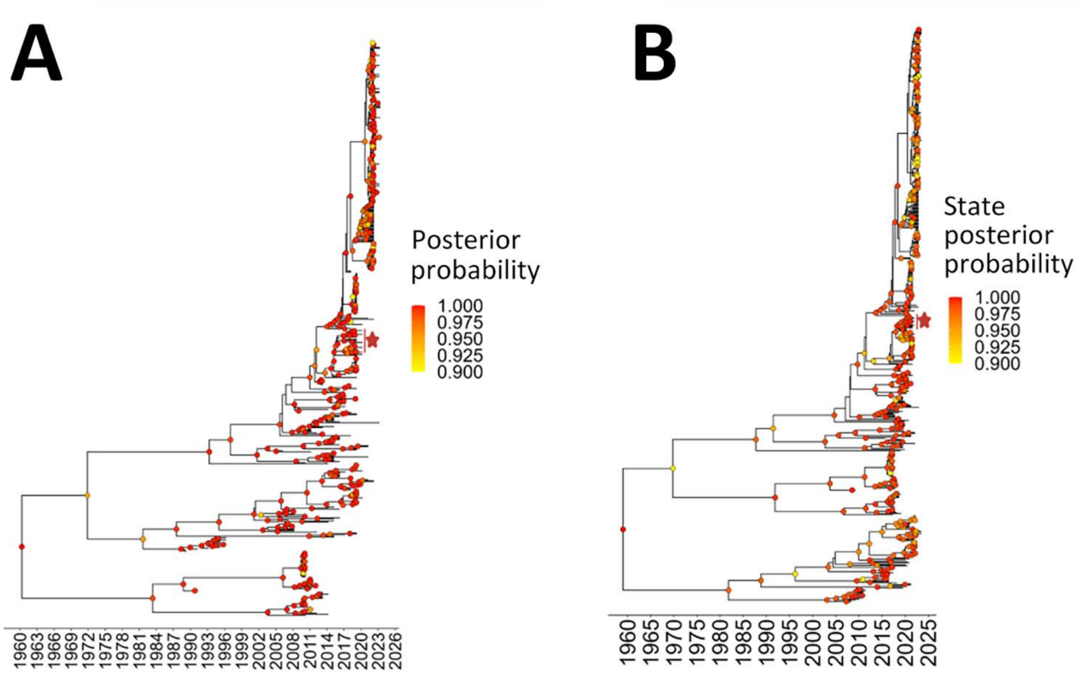

Appendix Figure 2. Time-scaled Bayesian inference of temporal (A) and phylogeographic (B) analyses of dengue virus serotype 3. Maximum clade credibility trees indicate node support determined by posterior probability (A) and state posterior probability (B). Node support values were scaled according to color from 0.9 (yellow) to 1.00 (red); values <0.90 are not displayed. Red stars indicate the specific clade where strains from Jamaica are located.
